# Differential expression of starch and sucrose metabolic genes linked to varying biomass yield in *Miscanthus* hybrids

**DOI:** 10.1186/s13068-021-01948-4

**Published:** 2021-04-19

**Authors:** Jose J. De Vega, Ned Peel, Sarah J. Purdy, Sarah Hawkins, Lain Donnison, Sarah Dyer, Kerrie Farrar

**Affiliations:** 1 Earlham Institute, Norwich, NR4 7UZ UK; 2 Institute of Biological, Environmental & Rural Sciences (IBERS), Aberystwyth University, Aberystwyth, SY23 3EE UK; 3 NSW Department of Primary Industries, Chief Scientist’s Branch, Locked Bag 21, Orange, NSW 2800 Australia; 4 NIAB, Cambridge, CB3 0LE UK

**Keywords:** Miscanthus, Starch, Sucrose, Yield, RNA-seq, Biomass, Transcriptional regulation, Co-expression network

## Abstract

**Background:**

*Miscanthus* is a commercial lignocellulosic biomass crop owing to its high biomass productivity and low chemical input requirements. Within an interspecific *Miscanthus* cross, progeny with high biomass yield were shown to have low concentrations of starch and sucrose but high concentrations of fructose. We performed a transcriptional RNA-seq analysis between selected *Miscanthus* hybrids with contrasting values for these phenotypes to clarify how these phenotypes are genetically controlled.

**Results:**

We observed that genes directly involved in the synthesis and degradation of starch and sucrose were down-regulated in high-yielding *Miscanthus* hybrids. At the same time, glycolysis and export of triose phosphates were up-regulated in high-yielding *Miscanthus* hybrids. These differentially expressed genes and biological functions were regulated by a well-connected network of less than 25 co-regulated transcription factors.

**Conclusions:**

Our results evidence a direct relationship between high expression of essential enzymatic genes in the starch and sucrose pathways and co-expression with their transcriptional regulators, with high starch concentrations and lower biomass production. The strong interconnectivity between gene expression and regulators, chemotype and agronomic traits opens the door to use the expression of well-characterised genes associated with carbohydrate metabolism, particularly in the starch and sucrose pathway, for the early selection of high biomass-yielding genotypes from large *Miscanthus* populations.

**Supplementary Information:**

The online version contains supplementary material available at 10.1186/s13068-021-01948-4.

## Background

*Miscanthus* is a candidate biofuel crop owing to its high biomass yield and low input requirements [1, 2]. It is also naturally adapted to a wide range of climate zones and land types [3, 4]. Currently, *Miscanthus* is mainly used for combustion, but there is keen interest in its development as a sustainable substrate for bioethanol or biomethane production [5, 6], as well as fibre products.

*Miscanthus* is a C4 perennial rhizomatous grass crop closely related to sugarcane (*Saccharum* spp.), sorghum (*S. bicolor*) and maize (*Zea mays*). However, unlike these species, *Miscanthus* is a non-food crop and can be grown on lower agricultural grade or marginal land to not compete with food production [7, 8].

Natural interspecific hybridisation events occur between several *Miscanthus* species with overlapping geographic distributions [9]. The main commercial *Miscanthus* genotype to date*, M.* x *giganteus*, is a sterile triploid hybrid resulting from the wild hybridisation between a diploid *M. sinensis* and a tetraploid *M. sacchariflorus. M.* x *giganteus* has desirable traits, including high yield and early establishment [10–12]. However, *M.* x *giganteus* must be clonally propagated, which doubles establishments costs compared to a seed-based option [13]. Therefore, several European breeding programmes are aiming to develop a seed-based crop through recreating the hybridisation event between *M. sinensis* and *M. sacchariflorus* to produce new hybrids that out-perform *M. xgiganteus* [13, 14].

*Miscanthus* is harvested for the structural cell wall polysaccharides, and as a result, multiple studies have focused on its structural carbohydrates [15, 16]. However, it is the processing and storage of non-structural carbohydrates (nsc), such as sucrose and starch, that underpin biomass traits [17].

We have previously shown that high-yielding *Miscanthus* genotypes from an interspecific hybrid mapping family had low starch concentrations in the stem and a low ratio of starch to fructose [17]. These distinctive carbohydrate profiles were consistent across years and growing environments; thus, the phenotype is likely to be genetically controlled [17, 18]. Unlike many C3 temperate grasses, C4 species such as *Miscanthus* or maize do not accumulate fructans, but instead accumulate starch as a transient form of storage carbohydrate [19, 20]. The concentration of starch in the mapping family was up to 15% of the dry weight (dw) on average. However, higher values were observed in the lowest yielding lines, raising the possibility of bred “starch-cane” *Miscanthus* for liquid biofuel or biogas generation [21]. Identifying differentially expressed genes (DEGs) that relate to the carbohydrate profile could further facilitate breeding for such traits.

In this study, we analysed root (all underground tissues, including both roots and rhizomes), stem and leaf RNA-seq data from the hybrid progeny from a cross of a diploid *M. sacchariflorus* genotype and a diploid *M. sinensis* genotype, which had contrasting carbohydrate profiles and yield measurements. We identified differentially expressed genes associated with the observed metabolic profiles using the recently completed *M. sinensis* reference genome (*Miscanthus sinensis* v7.1 DOE-JGI) [22]. Integrating expression and metabolic data is a logical strategy given the strong interconnectivity between genotype, chemotype and phenotype, and the lower genetic complexity of intermediate phenotypes, such as metabolites and yield subcomponents [23, 24].

## Results

### Contrasting carbohydrate metabolism in sequenced genotypes from a *Miscanthus* mapping family

**Fig. 1:**
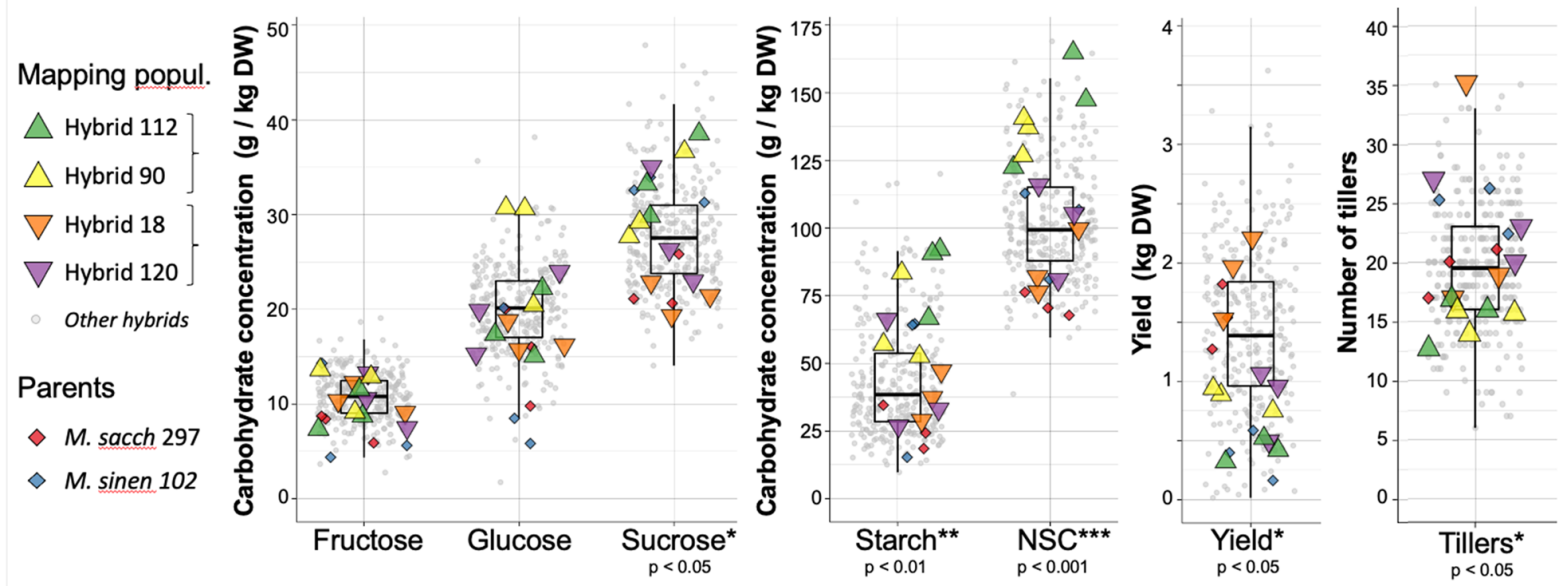
Concentrations of non-structural carbohydrates, number of tillers, and biomass yield in a mapping population comprised of 102 M*. sinensis* X *M. sacchariflorus* hybrids. Values for four hybrids with contrasting phenotypes (“high” and “low”), which were selected for RNA sequencing, are highlighted (Triangles). Significant differences (T-test) between the hybrids are annotated under each phenotype. The two parents of the family were also sequenced and phenotyped (diamonds). Boxplots summarise the distribution of values for the whole family for each phenotype

A total of 102 genotypes from a paired cross between diploid *M. sinensis* (“*M. sinen* 102”) and a diploid *M. sacchariflorus* (“*M. sacch* 297”) were established in field conditions and phenotyped. Non-structural carbohydrates were sampled in July 2014, during the summer growing season, and annual yield was obtained at harvest after the following winter. The distribution of carbohydrate concentrations and biomass yield for 98 hybrids were previously reported [17]. After including additional information about number of tillers and flowering day for the population (Fig. 1 and Additional file 6: Table S1), we observed significant correlations between number of tillers and starch (*r* = − 0.45, *p* < 0.001), fructose (*r* = 0.31, *p* < 0.005), and total NSC (*r* = − 0.40, *p* < 0.0001) for the whole family (Additional file 6: Table S1). We also observed significant correlations between number of tillers and the ratio of sucrose/starch (*r* = 0.37, *p* < 0.001), fructose/starch (*r* = − 0.45, *p* < 0.001), glucose/starch (*r* = − 0.38, *p* < 0.001) and sucrose/fructose (*r* = − 0.32, *p* < 0.01). We observed a significant positive correlation between biomass yield and the number of tillers (*r* = 0.62 ± 0.03 for three seasons, *p* < 0.001) in the progeny of this interspecific cross. We also observed a correlation between flowering day and yield (*r* = 0.37 ± 0.02 for two seasons, *p* < 0.001) and canopy weight (*r* = 0.47 ± 0.1 for two seasons, *p* < 0.001), but not with number of tillers (Additional file 6: Table S1). We also observed significant correlations between flowering day and fructose (*r* = − 0.37, *p* < 0.001) and the ratio of starch/fructose (*r* = − 0.37, *p* < 0.001). Genotypes initiating flowering (flag leaf visible) in August and September usually result in higher yield and height.

Four *M. sinensis* X *M. sacchariflorus* hybrids from this family (triangles in Fig. 1) were selected for RNA sequencing in their fourth growing season (2013), based on a higher or lower than the average number of tillers. The two parents of the family were also sequenced (diamonds in Fig. 1). When the four sequenced hybrids were divided into two groups (genotypes 112 and 90 against genotypes 18 and 120), we observed significant differences between these groups in the number of tillers (*p* < 0.05), biomass yield quantified as dry weight per plant (*p* < 0.05), and the final canopy heights (*p* < 0.05). We also observed a significant difference between these two groups in the concentrations of starch (*p* < 0.005) and sucrose (*p* < 0.05), but we did not observe significant differences between groups in the concentrations of fructose or glucose. The most significant difference (*p* < 0.001) was observed in the total concentration of non-structural carbohydrates (NSC), which was calculated as the sum of the glucose, fructose, sucrose and starch concentrations. We observed significant differences also in the fructose/starch (*p* < 0.05) and glucose/fructose ratios (*p* < 0.01). However, any other ratio between concentrations was not significantly different between the groups (Additional file 6: Table S2).

There was a significant difference between the *M. sacchariflorus* and *M. sinensis* parents in NSC (*p* < 0.05) and sucrose concentrations (*p* < 0.01). However, there was no significant difference between the parents in the starch, fructose or glucose concentrations (Additional file 6: Table S2). It is likely an example of heterosis (transgressive segregation) that significant differences in starch, fructose or glucose concentrations were observed in the hybrid progeny but not the parents.

### Differential expression (DE) analysis between hybrids and parental species

**Fig. 2:**
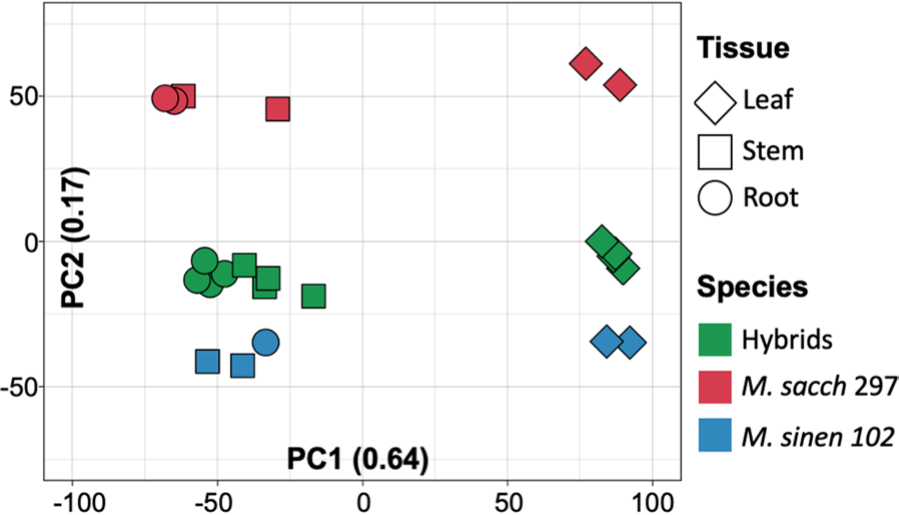
Principal component analysis of the normalised gene counts from 23 RNA-seq libraries generated from leaves (diamonds), stems (squares) and roots (circles) obtained from four *M. sinensis* X *M. sacchariflorus* hybrids (green shapes) with contrasting phenotypes and their parents (red and blue shapes). Gene counts were obtained from Kallisto alignments and normalised using DESeq2 for the top 1000 most variable genes

We performed RNA-seq from the leaf, stem and root (all underground tissues, including both roots and rhizomes) samples extracted from four *M. sacchariflorus* X *M. sinensis* interspecific hybrids, and their two parents (Table 1). When the normalised counts obtained from DESeq2 [25] were used to cluster the samples (Fig. 2), the samples firstly grouped by tissue (PC1) and secondly by species (PC2). As a result, the downstream analysis was performed for each tissue separately. Stem and root samples clustered together, and the clustering of these separately from the leaf tissue explained 64% of the variation. Species explains 17% of the variation, with the hybrids falling between the two parents, which were furthest apart from each other.

**Table 1:** RNA-seq libraries used in this study

Group	Tissue	Genotype	Library	%Aligns^a^	Covered transcripts^b^
High NSC/low yield/fewer tillers	Root	112	LIB2338	75.6	57,716
		90	LIB2341	76.6	55,950
	Stem	112	LIB2339	74.5	56,537
		90	LIB2342	75.4	59,459
	Leaf	112	LIB2340	75.7	54,316
		90	LIB2343	75.2	52,190
Low NSC/high yield/many tillers	Root	120	LIB2344	74.8	56,046
		18	LIB2347	73.2	59,909
	Stem	120	LIB2345	75.3	56,996
		18	LIB2348	76.7	56,875
	Leaf	120	LIB2346	74.2	56,948
		18	LIB2349	76.5	54,692
Progenitors	Leaf	297 *Msac*	LIB2353	70.1	50,288
		297 *Msac*	SAM1160	75.2	51,213
		102 *Msin*	LIB2351	78.3	53,304
		102 *Msin*	SAM1164	81.6	53,967
	Stem	297 *Msac*	LIB2352	69	54,192
		297 *Msac*	SAM1158	73.4	54,426
		102 *Msin*	LIB2350	79.9	56,797
		102 *Msin*	SAM1162	80.7	55,736
	Root	297 *Msac*	SAM1159	71.3	56,043
		297 *Msac*	SAM1161^c^	72.8	56,122
		102 *Msin*	SAM1163	74.2	59,043

Msac, M. sacchariflorus; Msin, M. sinensis; ^a^Proportion of reads that aligned in the reference; ^b^Transcripts with at least one mapping reads; ^c^root tip

**Fig. 3:**
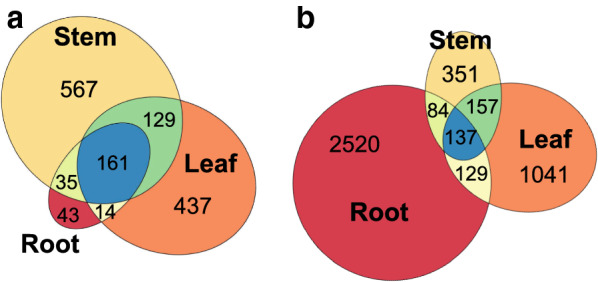
Number of differentially expressed genes shared between root, leaf and stem tissues among the “High NCS” and “Low NCS” *Miscanthus* hybrids at FDR < 0.05 (**a**), and between the hybrids and their progenitors (**b**). A gene only was considered DE between hybrids and parents when it was DE against both parents

We obtained 1386 differentially expressed genes (DEG; Additional file 6: Table S3) in total between the hybrids identified as “High NSC” and “Low NSC” (Fig. 1) at FDR < 0.05 (Fig. 3a). There were 892 DEGs in stems (598 up-regulated and 294 down-regulated), 741 DEGs in leaves (410 up-regulated and 331 down-regulated), and only 253 DEGs in roots (116 up-regulated and 137 down-regulated). 64% of the DEGs in roots were DE in both of the other tissues too, but most DEGs in stem or leaves were exclusively DE in either stem or leaves.

We also compared the expression between the hybrids against each parent and considered a gene as DE if it was DE in both comparisons at FDR < 0.05 (Additional file 6: Table S4). Under these criteria, there were 2870 DEGs in roots, 1,464 DEGs in leaves, and 729 DEGs in stems (Fig. 3b). Only 64 among these DEG were also DE between “High NSC” and “Low NSC” hybrids. There were 16,311 DEGs between the hybrids and *M. sinensis* alone (Additional file 1: Figure S1), and 15,616 DEGs between the hybrids and *M. sacchariflorus* alone (Additional file 2: Figure S2), this is over a third of the total transcriptome.

### Enriched Gene Ontology (GO) terms in DEGs

Enrichment analysis of GO terms over-represented among DE genes allowed us to identify the biological processes (BP) and molecular functions (MF) that are differentially regulated in each tissue. After annotating the reference transcriptome with the homologous proteins and full set of GO terms and (Additional file 6: Table S5), we simplified the results to the more general “GO slim” terms.

**Fig. 4:**
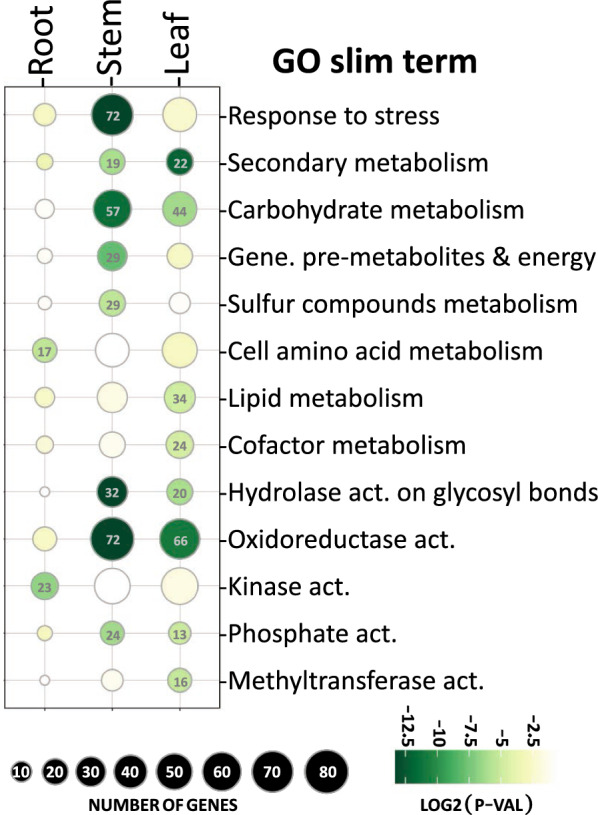
GO SLIM terms (rows) that were significantly enriched (*p* < 0.05) in each tissue (columns) among differentially expressed genes (DEG) from the “High NCS” and “Low NCS” *Miscanthus* hybrids DE analysis. The size of a bubble is proportional to the number of DEG annotated with that GO term. Rows are sorted by descending *p*-value (F-Fisher test) and the bubble colour is representative to the obtained p-value, from lower (dark green) to higher (light green). Yellow (*p* > 0.05) and white (*p* > 0.1) bubbles were not enriched. All the enriched GO SLIM terms for the “biological process” (top 8 rows) and “molecular function” (bottom 5 rows) GO categories were included

All the significant enrichment “GO slim” terms among DEGs between the “High NSC” and “Low NSC” hybrids were associated with metabolic processes, with the single exception of “response to stress” in stems (Fig. 4; Additional file 6: Table S6). Among the GO terms in the “biological process” category, the most significantly enriched ones (*p* < 0.001) were “Carbohydrate metabolism” and “Secondary metabolism” in stem and leaves, and “Generation of precursor metabolites and energy” and “response to stress” in stems. Among the “molecular process” category, “hydrolysis on glycosyl bonds” and “redox activities” were the most significantly enriched (*p* < 0.0001) in both stems and leaves (Additional file 6: Table S6).

Thirty-six enzymatic reactions were annotated among DEG in the stem (Table 2). Only six were down-regulated in “High NSC”; four involved in the generation of precursor metabolites and energy, namely 6-phosphofructokinase (EC 2.7.1.11) and Triose-phosphate isomerase (EC 5.3.1.1) in the glycolysis pathway; Malate dehydrogenase NADP(+) (EC 1.1.1.82) in the pyruvate metabolism; and 2-carboxy-D-arabinitol-1-phosphatase (EC 3.1.3.63); and one each in the other GO categories, namely Beta-N-acetylhexosaminidase (EC 3.2.1.52) and carboxypeptidase (EC 3.4.16.-).

**Table 2:** Thirty-eight differentially expressed genes highlighted in our analysis were involved in 19 reactions in the starch and sucrose metabolism and associated glycolysis reactions

GENE	Protein name	Enzyme name	Enzyme codE	ATH	Rice	Leaf FC	Stem FC	TF
Misin01G145100	BG8	Glucan endo-1,3-beta-D-glucosidase	3.2.1.39	1G64760	03g45390		2.22	11G251200
Misin01G337100	Beta-1,3-glucanase	Glucan endo-1,3-beta-D-glucosidase	3.2.1.39		03g25790	2.41		04G316000
Misin01G358800	SUS3	Sucrose synthase	2.4.1.13	4G02280	03g22120		1.98	
Misin02G115300	T11I18	Glucan endo-1,3-beta-D-glucosidase	3.2.1.39	3G04010	03g45390		1.99	12G221200 13G183300 15G100500
Misin02G205400	BAM1	Beta-amylase	3.2.1.2	3G23920	10g32810	2.36	2.37	
Misin02G326400	Beta-1,3-glucanase	Glucan endo-1,3-beta-D-glucosidase	3.2.1.39		03g25790	2.53		05G228900
Misin02G341300	Phosphoglycerate mutase	Phosphoglycerate mutase	5.4.2.11/.12		03g21260	2.45	4.35	
Misin03G195400	ISA3	Isoamylase	3.2.1.68	4G09020	09g29404	–	1.36	03G244700
Misin03G235900	TIM	Triose-phosphate isomerase	5.3.1.1	2G21170	09g36450		− 0.59	
Misin03G316100	HEXO2	Beta-N-acetylhexosaminidase	3.2.1.52	1G05590	07g38790	3.91		
Misin04G207500	AMY1	Alpha-amylase	3.2.1.1	4G25000		1.67		
Misin04G215400	ISA3	Isoamylase	3.2.1.68	4G09020	09g29404		1.07	04G236800
Misin04G312400		Beta-amylase	3.2.1.2				5.04	02G101700 11G195000
Misin05G335800	PHS2	Glycogen phosphorylase	2.4.1.1	3G46970	01g63270		1.07	
Misin06G202700	F15G16.1/SF10	Glucose-6-phosphate 1-epimerase	5.1.3.15	3G61610	01g46950	− 1.67		TF regulating 48 target genes
Misin06G358300	BGLU42/4	Beta-glucosidase	3.2.1.21	5G36890	01g67220	1.27		T131300
Misin07G322000	LSF1/SEX4	Isoamylase	3.2.1.68	3G01510	08g29160	− 1.02	–	
Misin07G352300	SBE2.2	1,4-Alpha-glucan branching enzyme	2.4.1.18	5G03650	02g32660		1.92	05G273100
Misin10G070300	SPS5	Sucrose-phosphate synthase	2.4.1.14		11g12810		1.1	03G157300 04G243500 04G398500 05G381500 07G307900
Misin11G067200	cwINV4/OsCIN2	Beta-fructofuranosidase	3.2.1.26	2G36190	04g33740		3.63	06G000800 15G053900
Misin11G111200	BGLU14	Beta-glucosidase	3.2.1.21	2G25630		− 1.26		
Misin11G121200	MLS	Malate synthase	2.3.3.9	5G03860	04g40990	5.07	5.71	12G063100
Misin11G141900	BGLU45/18	Beta-glucosidase	3.2.1.21	1G61810	04g43410	− 1.58		
Misin11G142000*	BGLU18	Beta-glucosidase	3.2.1.21		04g43410		1.21	
Misin12G113600	PFK2	6-Phosphofructokinase	2.7.1.11	5G47810	09g30240	− 6.94	− 6.65	01G471000 11G195000 18G256700
Misin12G147300	BGLU46	Beta-glucosidase	3.2.1.21	1G61820	04g43390		1.12	
Misin15G034600		Beta-amylase	3.2.1.2				1.09	04G230700
Misin16G118700	BG1	Glucan endo-1,3-beta-D-glucosidase	3.2.1.39	3G57270		− 3.26		
Misin17G123500	BG3	Glucan endo-1,3-beta-D-glucosidase	3.2.1.39	3G57240		− 2.79		
Misin17G131000	DBE1	Isoamylase	3.2.1.68	1G03310	05g32710		1.04	09G177000
Misin17G142700	HEXO3	Beta-N-acetylhexosaminidase	3.2.1.52	1G65590	05g34320		− 0.57	03G257000 07G206800 12G092100 16G048700
Misin17G216100	ALDH12A1	L-Glutamate gamma-semialdehyde dehydrogenase	1.2.1.88	5G62530		4.19	4.17	TF regulating five genes
Misin17G255500	AGPL3/APL3	Glucose-1-phosphate adenylyltransferase	2.7.7.27		05g50380		1.72	
Misin18G276400	Glycogen branching	1,4-Alpha-glucan branching enzyme	2.4.1.18				1.77	
Misin19G100900	SS2	Starch synthase	2.4.1.21		06g12450		2.44	
MisinT226600	BGL2	Glucan endo-1,3-beta-D-glucosidase	3.2.1.39	3G57260		2.45	4.22	
MisinT393000	SS3	Starch synthase	2.4.1.21	1G11720			1.63	T178900
MisinT552400	BAM1	Beta-amylase	3.2.1.2	3G23920	10g32810		3.03	T178900

Leaf/stem FC = Log2 fold-change expression “high NSC”/“Low NSC” hybrids in either lead or stem tissues; ATH/RICE = Homologous protein in Arabidopsis thaliana and rice (The prefixes “AT” or “LOC_Os” were excluded from the gene name). TF, Transcription factor regulating the gene, only TFs that were DE or which regulon was enriched in DE-targets are shown. The prefix “Misin” was excluded from the TF gene name

A similar analysis on the enriched GO slim terms among DEGs between hybrids and parents (Additional files 3, 6: Figure S3; Table S7) revealed that the most significantly enriched GO terms (*p* < 0.01) were in the root and associated with RNA/DNA binding and translation (including ribosome biogenesis and equivalent terms), and several biosynthetic processes. Remarkably, there were no enriched GO terms in the stem between hybrids and parents.

### DEG associated with the starch and sucrose metabolism

There were 88 DEGs associated with the enriched “Carbohydrate metabolism” (GO:5975) GO term (Additional file 6: Table S8), specifically 57 DEGs in stems (42 up-regulated and 15 down-regulated) and 44 DEGs in leaves (20 were up-regulated and 24 down-regulated). Thirteen DEGs were common to both tissues and showed close fold-change values in both tissues. All but two of these 88 DEGs could be functionally annotated, 52 and 56 of them had a homologous protein in *A. thaliana* or rice, respectively.

Twenty-nine DEGs were involved in enzymatic reactions that were part of the starch and sucrose metabolic pathways (KEGG pathway ath00500; Additional file 4: Figure S4). Among these, all 20 DEGs in stems were up-regulated in “High NSC”, but half of the DEGs in leaves (which were beta-glucosidases) were down-regulated in “High NSC”. Enzymatic proteins in the starch degradation pathway were DE in root and leaves (e.g. AMY3, ISA3, BAM1). At the same time, sucrose metabolism genes in the cytosol were only DE in stems (SUS3, SPS5). Similarly, reactions involving ADP-glucose were only DE in stems (e.g. AGP, SS2, SS3, SBE2).

Twenty-nine genes were annotated as involved in the “generation of precursor metabolites and energy” (GO:6091) (Additional file 6: Table S8), 17 of which could be annotated with an enzymatic code (KEGG pathway ath00010; Additional file 5: Figure S5). Six genes were involved in starch metabolism (ISA3, DBE1, PFK2, SBE2, PHS2). The phosphofructokinase 2 (PFK2) is the only one clearly down-regulated in “High NSC”. Among the others, a malate synthase (MLS) and an aldehyde dehydrogenase 12A1 involved in siRNAs generation, and an Fts protease (FTSH6) in the chloroplast were all highly up-regulated (FC > 5) in “High NSC”. On the other hand, triosephosphate isomerase (TIM) was down-regulated in “High NSC”.

**Fig. 5:**
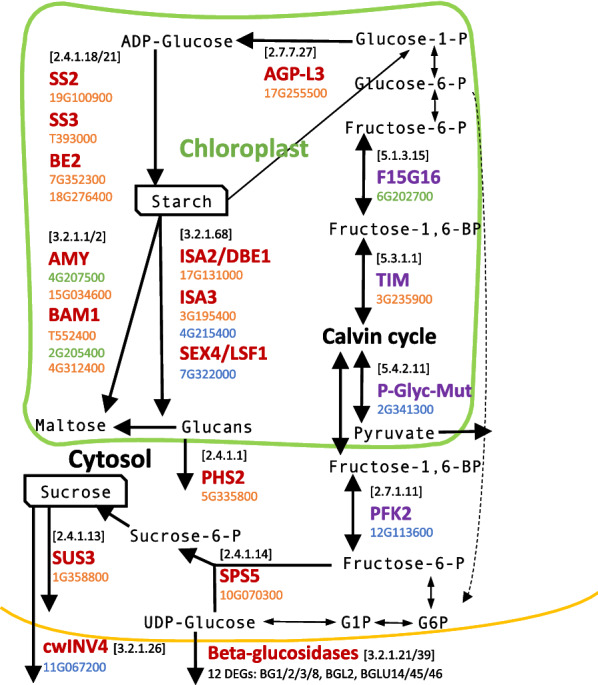
Schema of the starch and sucrose metabolism in plants, highlighting critical differentially expressed (DE) proteins between “High NSC” and “Low NSC” *Miscanthus* hybrids. Enzymatic codes are shown between brackets. DE *Miscanthus* genes are included under their respective protein (The prefix “Misin_” is not included in the gene name). Genes were differentially expressed in leaves (coloured in green), stems (orange) or both tissues (blue)

The relation between 32 DEGs involved in the 12 DE enzymatic reactions in starch and sucrose metabolism, plus three of the glycolysis reactions, are summarised in Fig. 5 and Tables 2 and 3.

**Table 3:** Additional carbohydrate and secondary metabolic enzymatic proteins highlighted in our differentially expression analysis between “high NSC” and “low NSC” *Miscanthus* hybrids

Gene	Enzyme name	Enzyme code	Leaf FC	stem FC	TF
Misin01G047600	Ent-copalyl diphosphate synthase	5.5.1.13	2.51		
Misin01G158200	Ent-isokaurene C2/C3-hydroxylase	1.14.13.143/14.112		1.83	
Misin01G349900	Indolin-2-one monooxygenase	1.14.13.138/14.157	− 4.68		
Misin01G390800	Mitogen-activated protein kinase	2.7.11.24		1.77	
Misin02G286600	Glutathione transferase	2.5.1.18		2.01	Misin02G101700
Misin02G293100	Glutathione transferase	2.5.1.18		4.15	Misin05G170700 Misin07G012200 Misin12G063100 Misin15G053900
Misin02G490600	Fucose-1-phosphate guanylyltransferase	2.7.7.30	1.84	2.9	
Misin03G233500	Beta-galactosidase	3.2.1.23	− 1.03		
Misin04G105800	Camalexin synthase	1.14.19.52	1.58		
Misin04G200300	Aldehyde dehydrogenase (NAD( +))	1.2.1.3	0.81		
Misin04G333300	Dimethylallyltranstransferase	2.5.1.1	− 1.4		Misin01G452600 Misin15G053900 It regulates 29 targets
Misin05G078900	Endo-1,4-beta-xylanase	3.2.1.8	− 2.78		
Misin05G312600	Trans-cinnamate 4-monooxygenase	1.14.14.91	− 2.11		
Misin06G200500	Pyruvate kinase	2.7.1.40	0.64		
Misin06G334400	Laccase	1.10.3.2	− 2.4		
Misin07G204200	Indolin-2-one monooxygenase	1.14.13.138/14.157	5.73		
Misin07G251100	6-phosphogluconolactonase	3.1.1.31	− 2.48		
Misin07G271500	Malate dehydrogenase (NADP( +))	1.1.1.82	− 3.12	− 0.85	Misin01G049500 Misin01G452600 Misin03G016900 Misin03G365800 Misin06G000800 Misin07G160300
Misin08G144100	Glycerophosphodiester phosphodiesterase	3.1.4.46	0.82		
Misin09G192700	Indolin-2-one monooxygenase	1.14.13.138/14.157	3.53		
Misin10G020200	2-carboxy-D-arabinitol-1-phosphatase	3.1.3.63	− 3.42	− 2.49	Misin01G019100 Misin01G049500 Misin06G000800 Misin07G160300
Misin10G067800	Non-reducing end alpha-L-arabinofuranosidase	3.2.1.55	− 1.95		Misin12G092100
Misin10G086500	Sugar-phosphatase	3.1.3.23	− 1.67		Misin07G251400.1
Misin11G031500	Nicotinamide N-methyltransferase	2.2.1.1		2.33	
Misin12G095900	Sinapoylglucose–malate O-sinapoyltransferase	2.3.1.92	2.55	3.49	
Misin15G165600	Ent-cassa-12,15-diene 11-hydroxylase	1.14.13.145/14.112	5.04	5.67	Misin05G245900 Misin08G171900 MisinT078100
Misin18G109800	Pyruvate dehydrogenase	1.2.4.1		4.21	TF regulating two genes
Misin19G207900	Indole-2-monooxygenase	1.14.14.153	1.52		
MisinT014600	3-hydroxyindolin-2-one monooxygenase	1.14.13.139/14.139	10.87	8.05	MisinT099300
MisinT014900	Indole-2-monooxygenase	1.14.14.153	11.4	5.99	MisinT099300
MisinT029700	Delta(24)-sterol reductase	1.3.1.72	2.52	1.5	Misin05G264600
MisinT167900	Alpha-galactosidase	3.2.1.22		0.81	
MisinT219600	Indolin-2-one monooxygenase	1.14.13.138/14.157	2.22	4.73	
MisinT258000	Glutathione transferase	2.5.1.18	2.57	1.55	
MisinT404400	Glutathione transferase	2.5.1.18	2.51	3.37	

Leaf FC or Stem FC = Log2 fold-change expression between “high NSC”/ “Low NSC” hybrids in either leaf or stem tissues. TF, Transcription factor regulating the gene, only TFs that were DE or which regulon was enriched in DE targets are shown

### DEG associated with other enriched GO terms

The 72 genes annotated as "Response to stress" were involved in a broad range of responses (Additional file 6: Table S10). On the other hand, the most significantly enriched GO terms in the "Molecular functions" category were associated with metabolic-related enzymatic reactions, namely “oxidoreductase activities” and “hydrolase activities”. The former included 38 cytochrome P450 proteins.

“Secondary metabolism” was enriched in both stems and leaves. 17 of the 19 DEGs in stems were up-regulated, but half of the DEGs in leaves were down-regulated. 16 of the 31 genes involved in the “secondary metabolism” were cytochrome P450 proteins (Additional file 6: Table S11). Six were included in benzoxazinoids biosynthesis, which is associated with defence in grasses. Another six were involved in terpenoids and phenylpropanoid biosynthesis (KEGG ath00900 and ath00940).

Many of the identified DEG in enriched functions showed no homologies in model organisms and consequently remain uncharacterised. This is the case in 36 DE genes involved in the carbohydrate metabolism (over 88 total), whose function was evidenced by the presence of a protein domain, but with an unclear role. A similar case is noted in two genes involved in the "generation of precursor metabolites", 12 genes involved in the “secondary metabolism”, and 17 genes involved in “response to stress”.

### Transcriptional regulatory co-expression network (TRN) inference and analysis of regulated target genes (regulons)

We annotated 5045 transcription factors (TFs) in the *M. sinensis* proteome based on homology with the Plant Transcription Factor Database (Additional file 6: Table S5) [26]. The set of target genes regulated by a given TF forms a *regulon*. We inferred the putative regulon of each TF based on co-expression between targets and TFs using the RTN package [27]. For 4,427 TFs we identified at least one target gene (Additional file 6: Table S12). The complete TRN included 26,710 genes (nodes) and 57,643 links between genes (edges).

We compared the overlap between the target genes in each regulon with the lists of DEGs previously obtained to identify regulons enriched in DE genes. We identified 100, 52 and 29 regulons (117 regulons in total) that were significantly enriched in DEGs in stems, leaves and roots, respectively (FDR < 0.05; Additional file 6: Table S13). Most regulons enriched in stems (62%) were only enriched in that tissue, but only 12% and 1.7% of the regulons in leaves and roots were exclusive to such tissue, respectively. We later verified this analysis using two-tail GSEA, including the expression fold-change to rank the genes (Additional file 6: Table S13).

On the other hand, we identified the GO terms enriched (FDR ≤ 0.05) in each regulon to clarify the processes it may be involved (Additional file 6: Table S14). 2,989 regulons were associated with at least one significant GO term. Among these, 213, 232, and 115 regulons were, respectively, annotated as involved in “carbohydrate metabolism” (GO:5975), “generation of precursor metabolites and energy” (GO:6091), and “secondary metabolism” (GO:19748); for a total of 515 unique regulons. The TFs regulating 12 of these 515 regulons were differentially expressed (Additional file 6: Table S14).

We identified 28 regulons (Table 4 and Additional file 6: Table S15) that were (i) enriched with the “carbohydrate metabolism”, “generation of precursor metabolites and energy”, or “secondary metabolism” GO terms; and were also (ii) enriched in DEGs, or where the TF was DE. These 28 regulons contained 806 target genes in total, but only 134 were DE (Additional file 6: Table S16).

**Table 4:** Regulons (set of genes regulated by a TF) significantly enriched in DEGs (or which TF was DE) and associated with “carbohydrate metabolism”, “generation of precursor metabolites and energy”, or “secondary metabolism” GO terms

TF	Regulon GO-EA	Regulon size	Regulon DE?	DE stem	DE leaf	DE root	TF DE?	TF description
Misin01G049500	Carb. Met	30	Yes	4	6	2	No	TSA1, tryptophan synthase alpha chain
Misin01G452600	Carb. Met	39	Yes	8	6	1	No	Homeodomain-leucine zipper transcription factor TaHDZipI-1
Misin02G383200	Carb. Met	21	Yes	4	0	0	No	LBD1, LOB domain-containing protein 1
Misin03G207100	Carb. Met	14	Yes	3	0	0	No	Beta-1,3-galactosyltransferase 7
Misin03G237200	Sec. met	19	No	0	2	0	Leaf	ODORANT1
Misin03G365800	Prec. Met	13	Yes	4	3	1	No	Putative MYB DNA-binding
Misin04G236800	Carb. Met & Prec. Met	35	Yes	4	1	0	No	TPA: putative HLH DNA-binding
Misin04G243500	Carb. Met	87	No	2	2	0	Leaf	Transcription factor bHLH137
Misin04G316000	Sec. met	17	No	0	3	0	Leaf	Transcription factor ABORTED MICROSPORES-like
Misin05G004600	Sec. met	18	No	2	0	0	Stem	WRKY transcription factor 31
Misin05G170700	Sec. met	23	Yes	4	0	0	No	Putative transcription factor bHLH041
Misin06G000800	Carb. Met & Prec. Met	24	Yes	6	8	2	Leaf/Stem/Root	TPA: putative HLH DNA-binding
Misin06G026100	Sec. met	22	Yes	6	0	0	No	Putative transcription factor bHLH041
Misin06G170200	Sec. met	19	No	3	0	0	Stem	Putative transcription factor bHLH041
Misin06G257300	Prec. Met	28	Yes	5	0	0	Stem	HSF1, heat stress transcription factor C-1b
Misin07G012200	Sec. met	29	Yes	4	0	0	No	Catalase
Misin07G160300	Prec. Met	11	Yes	3	2	1	No	RNA polymerase I termination factor isoform X1
Misin07G253100	Prec. Met	69	Yes	7	3	2	No	Hydroxymethylglutaryl-CoA synthase
Misin07G375400	Carb. Met & Prec. Met	31	No	3	2	0	Leaf	Ethylene-responsive transcription factor
Misin08G219000	Prec. Met	58	Yes	2	5	1	No	LAF1
Misin10G025900	Prec. Met	15	Yes	6	3	0	No	Light-inducible protein CPRF2
Misin12G063100	Carb. Met & Sec. Met	23	Yes	5	1	0	No	Ethylene-responsive transcription factor ERF113
Misin12G092100	Carb. Met	49	No	1	4	0	Leaf	Putative 12-oxophytodienoate reductase 11
Misin13G183300	Prec. Met	38	No	2	1	0	Stem	bHLH family
Misin15G053900	Sec. met	23	Yes	6	2	0	No	GLDP1, glycine dehydrogenase mitochondrial
Misin16G048700	Carb. Met	59	No	1	4	0	Leaf	MYB transcription factor
Misin18G114300	Prec. Met	3	No	0	1	0	Leaf	ODORANT1
MisinT099300	Sec. met	61	Yes	29	26	22	No	Pentatricopeptide repeat-containing

TF, Transcription factor; Regulon GO-EA, GO terms enriched in the target genes; Carb. Met., Carbohydrate metabolism (GO:5976); Sec. met., Secondary metabolism; Prec. Met., Generation of precursor metabolites and energy (GO:6091); Regulon size, Number of target genes in the regulon; Regulon DE? Is the regulon enriched in DE target genes?; DE stem/leaf/root, Number of target genes that are DE in stem/leaf/root, respectively; TF DE? Is the transcription factor DE in any tissue?

**Fig. 6:**
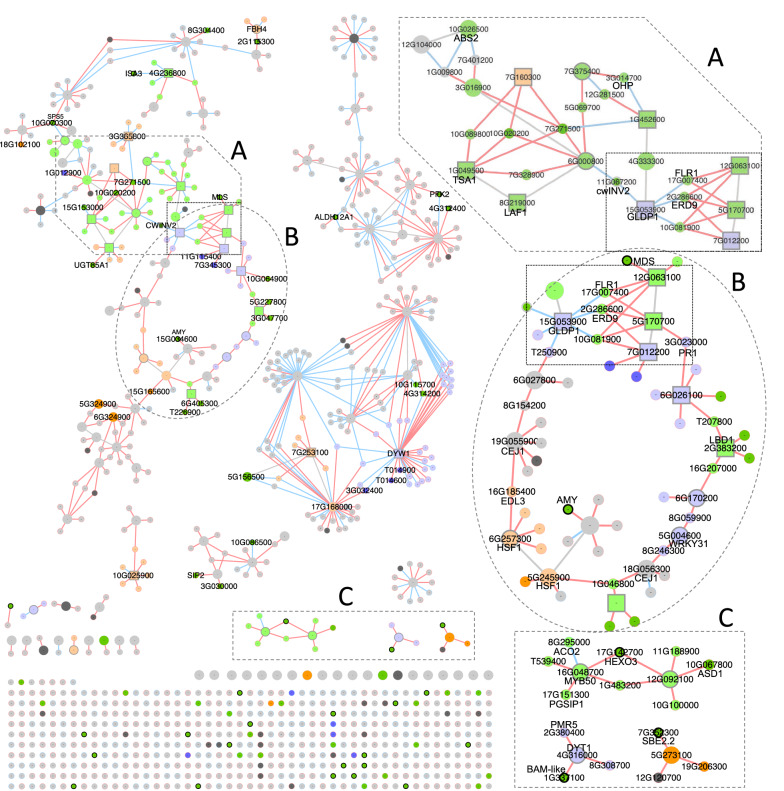
Transcriptional regulatory co-expression of all the differentially expressed genes (circles) observed between “High NSC” and “Low NSC” *Miscanthus* hybrids. Sixteen TFs which regulon was enriched in DEGs were also included (squares). TFs are represented in a larger size than the target genes. Nodes with a black or grey border are listed in Tables 2 or 4, respectively. Activation or repression between a TF and target gene are, respectively, represented by red or blue links (edges) between genes (nodes). Node colour corresponds to GO term enrichment, lighter colours for regulons and darker colours for individual genes; green: carbohydrate met., orange: generation of precursor met. and energy, purple/indigo: secondary met. At the bottom of the plot, DE target genes or TFs (larger size) in the TRN that were not connected to other DEGs

Figure 6 contains the transcriptional regulation co-expression network (TRN) for all the DEGs present in it, plus the 28 previously highlighted regulons (Table 3), coloured by enriched GO term. The TRN could be divided into one large, well-connected subnetwork, which included a highly co-regulated core (Fig. 6a) and a regulation loop (Fig. 6b). This subnetwork included 205 DEGs, and 20 of the 28 highlighted regulons (circles/squares with a dark grey border) in Table 3. It also included 5 of the target genes involved in starch metabolism in Table 2 (green circles with a black border in Fig. 6). This subnetwork was enriched in genes associated with “carbohydrate metabolism” (green nodes in Fig. 6). There were two other large subnetworks, but these were not clearly associated with the three GO terms. Three regulons (Fig. 6c) evidenced the co-regulation of several genes involved in carbohydrate metabolism, including starch and sucrose metabolism, by the same TF.

## Discussion

We performed a transcriptional RNA-seq analysis between selected *Miscanthus* hybrids with negative correlations between starch and sucrose concentrations and biomass yield.

Using a mapping family (*n* = 102) between a diploid *M. sinensis* and a diploid *M. sacchariflorus*, we previously demonstrated that high biomass-yielding *Miscanthus* hybrids had low starch and high fructose concentrations in the stem, and a lower ratio of sucrose, glucose and starch to fructose under peak growing conditions [17].

Here, we selected four hybrids from this mapping family based on the number of tillers (transect count), which was highlighted previously as a target phenotype for increasing biomass yield [28]. These four hybrids could be divided into two groups (Table 1), which showed significant differences in the concentrations of starch and sucrose, but not of hexose. The most significant differences were observed for total NSC because of the cumulative effect of the differences in starch and sucrose.

Tillering was correlated positively with yield and negatively with NSC, and flowering was correlated with yield and plant height. Flowering and senescence represent the termination of biomass accumulation and delayed flowering can result in increased biomass. However, not all late or non-flowering genotypes are high yielding, as was seen here. Likewise, while tillering is associated with yield in the progeny of this cross, the *M. sacchariflorus* parent had higher tillering than the sinensis parent, but very low biomass (Fig. 1). Additionally, a large number of progeny have a higher yield than both parents (Fig. 1), indicating that heterosis may well be a factor within the population.

Approximately 10% of the total genes in *Miscanthus* were differentially expressed (DE) between these two groups of hybrids in stems and leaves, but not many were in roots (Fig. 3a). Among these DE genes, there was an enrichment of genes involved in carbohydrate and secondary metabolism in stem and leaves, and in the “generation of precursor metabolites and energy” in stem only (Fig. 4). To better understand how these three biological processes are regulated, we built a transcriptional regulatory co-expression network (TRN; Fig. 6), which is later further discussed. However, these GO terms for biological processes were not regulated similarly in both tissues. While the DEGs in the enriched categories were predominantly up-regulated in stems, they were evenly up-regulated and down-regulated in leaves.

The DE of carbohydrate metabolising genes between the leaf, stem and root is to be expected as it has been previously reported that carbohydrates are differentially distributed between these tissues in *Miscanthus* in July, the same month our study was conducted [18, 29]. Specifically, starch was up to 6 × more concentrated in the leaves than the stems, the below-ground biomass preferentially accumulated starch, and soluble sugars tended to be in greater concentrations in the stems compared to leaves [18]. Our transcriptional observations therefore largely reflect the distribution of carbohydrates; starch metabolism transcripts were DE in the leaf where starch is the most abundant carbohydrate, and sucrose metabolising enzymes were DE in the predominantly sucrose accumulating stem [29]. Fewer DEGs were observed in roots. Seasonal carbohydrate profiling of rhizomes in four genotypes showed that the soluble sugar contents were similar between genotypes and across two sites located 340 km apart [29].

We observed that multiple genes involved in the synthesis (AGP, SS2, SS3, BE2) and degradation of starch in the chloroplast (AMY3, ISA3, SEX4, BAM1) were down-regulated in high biomass-yielding genotypes (Fig. 5; Table 2). We also observed down-regulation of genes involved in the synthesis (SPS5) and degradation (SUS) of sucrose in high biomass-yielding genotypes. Genes involved in the starch metabolic pathway are up-regulated by a high sugar status [30–32], as there was a negative relationship between yield and soluble sugar (i.e. high yielders had lower sugar), it is consistent that the expression of sugar stimulated genes would be lower in high-yielding genotypes.

Contrary to this, we noticed the upregulation with a high fold-change in high biomass yield genotypes of triosephosphate isomerase TIM/PDTPI, which encodes a plastidic triose phosphate isomerase [33], and Phosphofructokinase 2 (PFK2). PFK2 catalyses the penultimate step before usable energy is extracted from the phosphorylated products of photosynthesis. This enzyme is, therefore, a main control point of glycolysis. The observation that high biomass plants have low carbohydrates can seem counter-intuitive, but the rationale is highly logical; high biomass plants maximise growth at the expense of their carbon reserves [34], whereas slow-growing types accumulate their reserves. The upregulation of the PFK2 gene encoding a major glycolytic enzyme is suggestive of a more rapid metabolism of photosynthate to fuel growth in the high-yielding types. In summary, starch and sucrose synthesis was down-regulated in high-yielding *Miscanthus* hybrids, while glycolysis and export of triose phosphates was up-regulated in high-yielding *Miscanthus* hybrids.

These results support that high-yielding *Miscanthus* genotypes were more rapidly accumulating structural mass, likely cellulose via sucrose metabolism [35–37], at the expense of starch [17, 29, 38]. The latter is further supported by the significant differences in the fructose-to-starch (but not glucose-to-starch) ratio between high and low yielding hybrids [17], which was also observed between the sequenced hybrids. Fructose is an indicator of sucrose metabolism, because it is produced exclusively from the metabolism of sucrose by the action of sucrose synthases (SUS), while glucose is produced by the metabolism of both sucrose and starch [39, 40]. Furthermore, in a C13 labelling experiment, it was observed that a greater proportion of the labelled carbon was observed in the insoluble fraction (mainly comprising cellulose) of a rapidly growing *Miscanthus* genotype, whereas a greater proportion was partitioned into starch in a slower-growing type [17]. Our results, therefore, add to these previous observations with the addition of transcriptomic evidence of the relationship between carbon metabolism, partitioning and growth.

We built a transcriptional regulatory co-expression network (TRN) that included 4,427 regulons. We identified the 28 regulons (Table 4) that were significantly enriched in the “carbohydrate metabolism”, “generation of precursor metabolites and energy”, or “secondary metabolism” GO terms, and also in DE target genes (or where the TF itself was a DEG).

When we plotted the TRN for every DEGs plus these 28 regulons (Fig. 6), 20 of these 28 regulons were well connected to each other’s and formed a contained subnetwork with a “core” (Fig. 6a) and a “loop” (Fig. 6b). At the core, and TF 6G000800 was DE in every tissue and regulated six genes, including a Beta-fructofuranosidase (Table 2), a Malate dehydrogenase (Table 3), a 2-carboxy-D-arabinitol-1-phosphatase (Table 3), and an uncharacterised gene (07G271500). This gene (07G271500) was remarkably co-regulated by six different TFs. TFs GLDP1 (15G053900) and bHLH041-like (5G170700) appeared to be the link between the core and loop of the subnetwork. The two transcription factors were connected to FLR1, ERD9 and indirectly to the highly expressed a malate transferase (MLS).

The loop (Fig. 6b) included 14 TFs that were co-regulated and activated. These TF were connected through single target genes that were not well characterised. The other two large subnetworks were not clearly associated to the studied GO terms. One of them included, however, two of the 28 highlighted regulons (DYW1 and 7G253100). Three small subnetworks with just one/two TFs highlighted the co-expression of several targeted genes (Fig. 6c).

## Conclusion

Our results evidence a direct relationship between high expression of essential enzymatic genes in the starch and sucrose synthesis pathway, their transcriptional regulators (TFs) and co-expression, high starch concentrations, and lower biomass production. The strong interconnectivity between expression, regulators, genotype, chemotype and agronomic traits opens the door to use the expression of well-characterised genes associated with carbohydrate metabolism, particularly in the starch and sucrose pathway, for the early selection of high biomass-yielding genotypes from large *Miscanthus* populations. Since regulation appears to play a key role in NSC content, these identified TFs offer a breeding or biotechnological target for improvement, or selection of “starch-rich” genotypes.

## Methods

### Mapping population establishment and phenotyping

A total of 102 genotypes from a paired cross between diploid *M. sinensis* genotype “*M. sinen* 102” and a diploid *M. sacchariflorus* genotype “*M. sacch* 297” were sown from seed in trays in a glasshouse in 2009. In 2010, individual plants were split to form three replicates of each genotype and then planted out into the field in a spaced-plant randomised block design comprising three replicate blocks at IBERS, Aberystwyth, UK. Tiller numbers were counted in the field in 2012. Plant material was harvested for RNA extraction from additional clonal pot grown plants in an unheated glasshouse mid-May 2012, when plants were actively growing. Thus, leaves were fully expanded, stems were elongating, and no flowering had occurred. Plants were checked for the presence of rhizome prior to final selections being made, and only those with rhizome present were selected for this study. Details of the carbohydrate analysis were previously described [17]. Briefly, the family was harvested in February 2015 following the 2014 growing season. Biomass was dried to a constant weight, and the average DW weight per plant (kg) was calculated. Soluble sugars were extracted and quantified enzymatically and photometrically from known standard curves on the same plate, as previously detailed [29]. Starch was extracted using a modified *Megazyme* commercial assay procedure and quantified photometrically from known standard curves on the same plate, as previously described [29]. Four hybrid genotypes were selected based on a low or high number of tillers (transect count of tillers). Correlation between concentrations, plant height and biomass phenotypes for the whole mapping population was previously quantified [17]. Pearson’s correlation values between the number of tillers and the other phenotypes were determined for the whole family. Differences between the four selected hybrids for all phenotypes were determined with Student’s two-tailed t-tests.

### RNA sequencing and pre-processing

RNA was extracted from the four selected hybrids, as well as from the two parents of the family. Extraction was performed using RNeasy Plant Mini kit (Qiagen, CA, USA) according to the manufacturer’s instructions. Total RNA samples were sent to the sequencing service at the Earlham Institute (Norwich, UK) where standard Illumina RNA-seq libraries were prepared and sequenced using the HiSeq 2000 platform. The raw reads were filtered with Trim Galore [41] using the default options for paired-end reads to remove Illumina adaptor sequences and reads with quality scores below 20. Cleaned reads were aligned to the *M. sinensis* reference genome (*Miscanthus sinensis* v7.1 DOE-JGI, http://phytozome.jgi.doe.gov) [22] downloaded from Phytozome with Kallisto using the “quant” mode with default options [42]. Previously, the reference was indexed using the *M. sinensis* gene annotation (*Miscanthus sinensis* v7.1 DOE-JGI, http://phytozome.jgi.doe.gov) downloaded from Phytozome in GFF3 format. This same gene annotation was functionally annotated with GO terms and enzyme codes with the command-line version of Blast2GO [43] using BLASTX with an E-value of 1e−10 and the NCBI non-redundant (nr) and EBI InterPro databases. To identify and annotate the transcription factors*,* the *M. sinensis* proteome was downloaded from Phytozome and aligned to the Plant Transcription Factor Database (version 5) [26] using Diamond [44]. We retained alignments with a minimum identify of 70% and score of 200.

### Differential expression and enrichment analysis

The differential expression and enrichment analysis are fully available in an R notebook (See Data availability), which is also available via Github. Briefly, Kallisto count files, one from each of the 23 libraries, were imported in R using TXimport [45]. Differential analysis was performed using DESeq2 [25] for each tissue (root, stem, leaf) independently. Raw gene counts were obtained from Kallisto alignments and normalised using DESeq2 for the top 1,000 most variable genes to cluster the samples. Genes with a False Discovery Rate (FDR) < 0.05 were considered differentially expressed (DE). We compared two groups of hybrids; each hybrid group was composed of two genotypes (genotypes 112 and 90 against genotypes 18 and 120). We also compared the hybrids against the *M. sacchariflorus* and *M. sinensis* parent, one at the time. A gene only was considered DE between hybrids and parents when it was DE against both parents. The enrichment analysis was based on an F-Fisher test (FDR < 0.05) using the library topGO [46] with the “weight01” algorithm. Using the lists of DE genes and functional annotation as inputs, topGO compared the number of DEGs in each category with the expected number of genes for the whole transcriptome. The “weight01” algorithm resolves the relations between related GO ontology terms at different levels. Enriched categories were plotted using ggplot2 [47]. Genes in enriched GO terms were further analysed using the online Phytomine [48] and Thalemine [49] databases. Genes annotated with enzyme codes were plotted using the online KEGG mapper [50].

### Transcriptional regulatory network (TRN) inference and regulon enrichment analysis

The TRN inference and regulon enrichment analysis was done with RTN v. 2.10.1 [27] and topGO v. 2.38.1 [46]. The code is fully available in an R notebook (see Data availability), which is also available via Github. Briefly, the normalise counts from the previous analysis with DESeq2 and the list of known TFs in *M. sinensis* were provided to the RTN package with default options. RTN uses permutation (1000 permutations, FDR < 0.05) to remove non-significant TF-target associations, and bootstrapping (100 bootstraps, 95% consensus) to remove unstable interactions, before applying the ARACNE algorithm (eps ≥ 0) for network reconstruction. GO term enrichment in regulons with topGO was done as previously described but using the list of target genes in a given regulon as gene-set instead of the list of DEGs. The overlap between the target genes in each regulon to the lists of DEGs was done by enrichment analysis with Master Regulatory Analysis (MRA) and two-tail GSEA. MRA compared the list of DEGs (present/absent) with the members in each regulon. GSEA ranked the genes by expression fold-change and compared with either the activated (positive regulon) and repressed (negative regulon) subsets of each regulon. Both methods are implemented in the RTN package and were run with default options (except minimum regulon size of 5) for each of the tissues in the two studies: low against high NSC content hybrids, or hybrids against parents (heterosis). An igraph object was generated with RTN, exported to an xgmml file and imported into Cytoscape (v. 3.8.2) to plot the Network.

## Supplementary Information

**Additional file 1: Figure S1.** Number of differentially expressed genes shared between root, leaf and stem tissues between the hybrids and the *M. sinensis* progenitor.

**Additional file 2: Figure S2.** Number of differentially expressed genes shared between root, leaf and stem tissues between the hybrids and the *M. sacchariflorus* progenitor.

**Additional file 3: Figure S3.** GO SLIM terms (rows) that were significantly enriched (p < 0.05) in each tissue (columns) among differentially expressed genes (DEG) from the expression analysis between the hybrids and both progenitors. The size of a bubble is proportional to the number of DEG annotated with that GO term. Rows are sorted by descending p-value (F-Fisher test) and the bubble colour is representative to the obtained p-value, from lower (dark green) to higher (light green). Yellow (p > 0.05) and white (p > 0.1) bubbles were not enriched. All the enriched GO SLIM terms for the “biological process” (top 8 rows) and “molecular function” (bottom 5 rows) GO categories were included.

**Additional file 4: Figure S4.** Down-regulated enzymatic reactions in the “starch and sucrose metabolism” pathway from KEGG (KEGG pathway ath00500) that were down-regulated in “high NSC” hybrids, which had higher concentrations of starch and sucrose.

**Additional file 5: Figure S5**. Enzymatic reactions in the “glycolysis/gluconeogenesis” pathway from KEGG (KEGG pathway ath00010) that were down-regulated (red boxes) or up-regulated (green boxes) in “high NSC” hybrids, which had higher concentrations of starch and sucrose.

**Additional file 6: Table S1.** Individual trait scores and Person correlation between traits. **Table S2.** Traits significantly different (T-test) between the sequenced samples. **Table S3.** Normalised counts, expression fold-change and P-values for all the genes in roots, stem and leaf tissue between groups of hybrids. **Table S4.** Normalised counts, expression fold-change and P-values for all the genes in roots, stem and leaf tissue between hybrids and parents. **Table S5.** Functional annotation, GO and enzyme codes for all the genes in the reference genome. **Table S6.** Enriched GO terms among DEG between groups of hybrids. **Table S7.** Enriched GO terms among DEG between hybrids and parents. **Table S8.** Detailed functional annotation of 88 DEG within the enriched “carbohydrate metabolism” GO term. **Table S9.** Detailed functional annotation of 29 DEG within the enriched “generation of precursor metabolites and energy” GO term. **Table S10.** Detailed functional annotation of 72 DEG within the enriched “response to stress” GO term. **Table S11.** Detailed functional annotation of 31 DEG within the enriched “secondary metabolism” GO term. **Table S12.** Transcription factor and target genes contained in each regulon in the TRN. **Table S13.** Enrichment analysis of differential expression genes for each regulon in the TRN. **Table S14.** Enrichment analysis of GO terms in each regulon for the TRN. **Table S15.** Annotation of the twenty-eight regulons were enriched with the “carbohydrate metabolism”, “generation of precursor metabolites and energy”, or “secondary metabolism” GO terms; and were also enriched in DEGs, or where the TF was DE. **Table S16.** Annotation of the target genes in the twenty-eight highlighted regulons.

## Data Availability

Raw reads are deposited in the Short Reads Archive (SRA) under Bioproject ID PRJNA639832. The R code used in the differential expression and enrichment analysis was deposited in Zenodo (http://doi.org/10.5281/zenodo.3834007) and Github (https://github.com/jjdevega/miscanthus_starch_rnaseq). The R code used in the Transcriptional Regulation co-expression analysis was deposited in Github (https://github.com/jjdevega/miscanthus_transcriptional_regulatory_coexpression_network).

## Abbreviations

BP: Biological processes (Gene ontology category)
DE: Differentially expressed
DEG: Differentially expressed gene
DW: Dry weight
EC: Enzyme code
FDR: False Discovery Rate
GO: Gene ontology
KEGG: Kyoto Encyclopedia of Genes and Genomes
MF: Molecular function (Gene ontology category)
NADP: Nicotinamide adenine dinucleotide phosphate
NSC: Non-structural carbohydrates
PC: Principal component
*r*: Pearson correlation
RNA-seq: RNA next-generation sequencing
siRNA: Small interference RNA

## References

[1] Somerville C, Youngs H, Taylor C, Davis SC, Long SP (2010). Feedstocks for lignocellulosic biofuels. Science.

[2] Donnison IS, Fraser MD (2016). Diversification and use of bioenergy to maintain future grasslands. Food Energy Security.

[3] Heaton EA, Dohleman FG, Miguez AF, Juvik JA, Lozovaya V, Widholm J, Zabotina OA, McIsaac GF, David MB, Voigt TB. *Miscanthus*: a promising biomass crop. In: Advances in Botanical Research, 56; 2010; pp. 75–137.

[4] Hodkinson TR, Chase MW, Lledó DM, Salamin N, Renvoize SA (2002). Phylogenetics of *Miscanthus*, *Saccharum* and related genera (Saccharinae, Andropogoneae, Poaceae) based on DNA sequences from ITS nuclear ribosomal DNA and plastid trnL intron and trnL-F intergenic spacers. J Plant Res.

[5] Kam J, Thomas D, Pierre S, Ashman C, McCalmont JP, Purdy SJ. A new carbohydrate retaining variety of *Miscanthus* increases biogas methane yields compared to *M*. x *giganteus* and narrows the yield advantage of maize. Food Energy Security*.* 2020, e224.

[6] Kiesel A, Wagner M, Lewandowski I (2017). Environmental performance of *Miscanthus*, switchgrass and maize: Can C4 perennials increase the sustainability of biogas production?. Sustainability.

[7] Wagner M, Mangold A, Lask J, Kiesel A, Lewandowski I (2018). Economic and environmental performance of *Miscanthus* cultivated on marginal land for biogas production. GCB Bioenergy.

[8] McCalmont JP, Hastings A, McNamara NP, Richter GM, Robson P, Donnison IS, Clifton-Brown J (2017). Environmental costs and benefits of growing *Miscanthus* for bioenergy in the UK. GCB Bioenergy.

[9] Hodkinson T, Klaas M, Jones M, Prickett R, Barth S (2015). *Miscanthus*: a case study for the utilization of natural genetic variation. Plant Genetic Res.

[10] Zub HW, Brancourt-Hulmel M (2010). Agronomic and physiological performances of different species of *Miscanthus*, a major energy crop. A review. Agronomy Sustain Dev.

[11] Christian D, Riche A, Yates N (2008). Growth, yield and mineral content of *Miscanthus* x *giganteus* grown as a biofuel for 14 successive harvests. Ind Crops Prod.

[12] Davey CL, Jones LE, Squance M, Purdy SJ, Maddison AL, Cunniff J, Donnison I, Clifton-Brown J (2017). Radiation capture and conversion efficiencies of *Miscanthus sacchariflorus*, *M. sinensis* and their naturally occurring hybrid *M. x giganteus*. GCB Bioenergy..

[13] Hastings A, Mos M, Yesufu JA, McCalmont J, Schwarz K, Shafei R, Ashman C, Nunn C, Schuele H, Cosentino S (2017). Economic and environmental assessment of seed and rhizome propagated *Miscanthus* in the UK. Front Plant Sci.

[14] Arnoult S, Brancourt-Hulmel M (2015). A review on miscanthus biomass production and composition for bioenergy use: genotypic and environmental variability and implications for breeding. BioEnergy Res.

[15] Van der Weijde T, Kiesel A, Iqbal Y, Muylle H, Dolstra O, Visser RG, Lewandowski I, Trindade LM (2017). Evaluation of *Miscanthus sinensis* biomass quality as feedstock for conversion into different bioenergy products. GCB Bioenergy.

[16] da Costa RM, Pattathil S, Avci U, Winters A, Hahn MG, Bosch M (2019). Desirable plant cell wall traits for higher-quality miscanthus lignocellulosic biomass. Biotechnol Biofuels.

[17] Maddison AL, Camargo-Rodriguez A, Scott IM, Jones CM, Elias DMO, Hawkins S, Massey A, Clifton-Brown J, McNamara NP, Donnison IS (2017). Predicting future biomass yield in *Miscanthus* using the carbohydrate metabolic profile as a biomarker. GCB Bioenergy.

[18] Purdy SJ, Maddison AL, Cunniff J, Donnison I, Clifton-Brown J (2015). Non-structural carbohydrate profiles and ratios between soluble sugars and starch serve as indicators of productivity for a bioenergy grass. AoB Plants..

[19] De Souza AP, Arundale RA, Dohleman FG, Long SP, Buckeridge MS (2013). Will the exceptional productivity of *Miscanthus* x *giganteus* increase further under rising atmospheric CO2?. Agric For Meteorol.

[20] Miyake H (2016). Starch accumulation in the bundle sheaths of C3 plants: a possible pre-condition for C4 photosynthesis. Plant Cell Physiol.

[21] Purdy SJ, Maddison AL, Nunn CP, Winters A, Timms-Taravella E, Jones CM, Clifton-Brown JC, Donnison IS, Gallagher JA (2017). Could Miscanthus replace maize as the preferred substrate for anaerobic digestion in the United Kingdom? Future breeding strategies. GCB Bioenergy.

[22] Mitros T, Session AM, James BT, Wu GA, Belaffif MB, Clark LV, Shu S, Dong H, Barling A, Holmes JR (2020). Genome biology of the paleotetraploid perennial biomass crop *Miscanthus*. Nat Commun.

[23] Keurentjes JJ (2009). Genetical metabolomics: closing in on phenotypes. Curr Opin Plant Biol.

[24] Riedelsheimer C, Czedik-Eysenberg A, Grieder C, Lisec J, Technow F, Sulpice R, Altmann T, Stitt M, Willmitzer L, Melchinger AE (2012). Genomic and metabolic prediction of complex heterotic traits in hybrid maize. Nat Genet.

[25] Love MI, Huber W, Anders S (2014). Moderated estimation of fold change and dispersion for RNA-seq data with DESeq2. Genome Biol.

[26] Jin J, Tian F, Yang D-C, Meng Y-Q, Kong L, Luo J, Gao G. PlantTFDB 4.0: toward a central hub for transcription factors and regulatory interactions in plants. Nucleic Acids Res. 2016; gkw982. 10.1093/nar/gkw982 PMC5210657 27924042

[27] Fletcher MN, Castro MA, Wang X, De Santiago I, O’Reilly M, Chin S-F, Rueda OM, Caldas C, Ponder BA, Markowetz F (2013). Master regulators of FGFR2 signalling and breast cancer risk. Nat Commun.

[28] Robson P, Jensen E, Hawkins S, White SR, Kenobi K, Clifton-Brown J, Donnison I, Farrar K (2013). Accelerating the domestication of a bioenergy crop: identifying and modelling morphological targets for sustainable yield increase in *Miscanthus*. J Exp Bot.

[29] Purdy SJ, Cunniff J, Maddison AL, Jones LE, Barraclough T, Castle M, Davey CL, Jones CM, Shield I, Gallagher J (2015). Seasonal carbohydrate dynamics and climatic regulation of senescence in the perennial grass, *Miscanthus*. BioEnergy Res.

[30] Mita S, Suzuki-Fujii K, Nakamura K (1995). Sugar-inducible expression of a gene for [beta]-amylase in *Arabidopsis thaliana*. Plant Physiol.

[31] Nakamura K, Ohto M-A, Yoshida N, Nakamura K (1991). Sucrose-induced accumulation of β-amylase occurs concomitant with the accumulation of starch and sporamin in leaf-petiole cuttings of sweet potato. Plant Physiol..

[32] Price J, Laxmi A, Martin SKS, Jang J-C (2004). Global transcription profiling reveals multiple sugar signal transduction mechanisms in *Arabidopsis*. Plant Cell.

[33] Chen M, Thelen JJ (2010). The plastid isoform of triose phosphate isomerase is required for the postgerminative transition from heterotrophic to autotrophic growth in *Arabidopsis*. Plant Cell.

[34] Sulpice R, Pyl E-T, Ishihara H, Trenkamp S, Steinfath M, Witucka-Wall H, Gibon Y, Usadel B, Poree F, Piques MC (2009). Starch as a major integrator in the regulation of plant growth. Proc Natl Acad Sci.

[35] Coleman HD, Yan J, Mansfield SD (2009). Sucrose synthase affects carbon partitioning to increase cellulose production and altered cell wall ultrastructure. Proc Natl Acad Sci.

[36] Mood SH, Golfeshan AH, Tabatabaei M, Jouzani GS, Najafi GH, Gholami M, Ardjmand M (2013). Lignocellulosic biomass to bioethanol, a comprehensive review with a focus on pretreatment. Renew Sustain Energy Rev.

[37] McFarlane HE, Döring A, Persson S (2014). The cell biology of cellulose synthesis. Annu Rev Plant Biol.

[38] Lloyd JR, Kossmann J (1930). Starch trek: the search for yield. Front Plant Sci.

[39] Smith AM, Zeeman SC, Smith SM (2005). Starch degradation. Annu Rev Plant Biol.

[40] Koch K (2004). Sucrose metabolism: regulatory mechanisms and pivotal roles in sugar sensing and plant development. Curr Opin Plant Biol.

[41] Krueger F. Trim galore. 2020: https://github.com/FelixKrueger/TrimGalore

[42] Bray NL, Pimentel H, Melsted P, Pachter L (2016). Near-optimal probabilistic RNA-seq quantification. Nat Biotechnol.

[43] Conesa A, Götz S, García-Gómez JM, Terol J, Talón M, Robles M (2005). Blast2GO: a universal tool for annotation, visualization and analysis in functional genomics research. Bioinformatics.

[44] Buchfink B, Xie C, Huson DH (2015). Fast and sensitive protein alignment using DIAMOND. Nat Methods.

[45] Soneson C, Love MI, Robinson MD (2015). Differential analyses for RNA-seq: transcript-level estimates improve gene-level inferences. F1000Res.

[46] Alexa A, Rahnenfuhrer J. topGO: enrichment analysis for gene ontology. R package version 2.42.0. 2010.

[47] Wickham H: ggplot2: elegant graphics for data analysis: Springer; 2016.

[48] Goodstein DM, Shu S, Howson R, Neupane R, Hayes RD, Fazo J, Mitros T, Dirks W, Hellsten U, Putnam N (2012). Phytozome: a comparative platform for green plant genomics. Nucleic Acids Res.

[49] Krishnakumar V, Hanlon MR, Contrino S, Ferlanti ES, Karamycheva S, Kim M, Rosen BD, Cheng C-Y, Moreira W, Mock SA (2015). Araport: the *Arabidopsis* information portal. Nucleic Acids Res.

[50] Kanehisa M, Sato Y (2020). KEGG Mapper for inferring cellular functions from protein sequences. Protein Sci.

