# Supplementary figures and images for: Differential expression of starch and sucrose metabolic genes linked to varying biomass yield in Miscanthus hybrids
Source: Biotechnol Biofuels. 2021 Apr 19;14:98. doi: 10.1186/s13068-021-01948-4 (PMC8056674; doi:10.1186/s13068-021-01948-4)

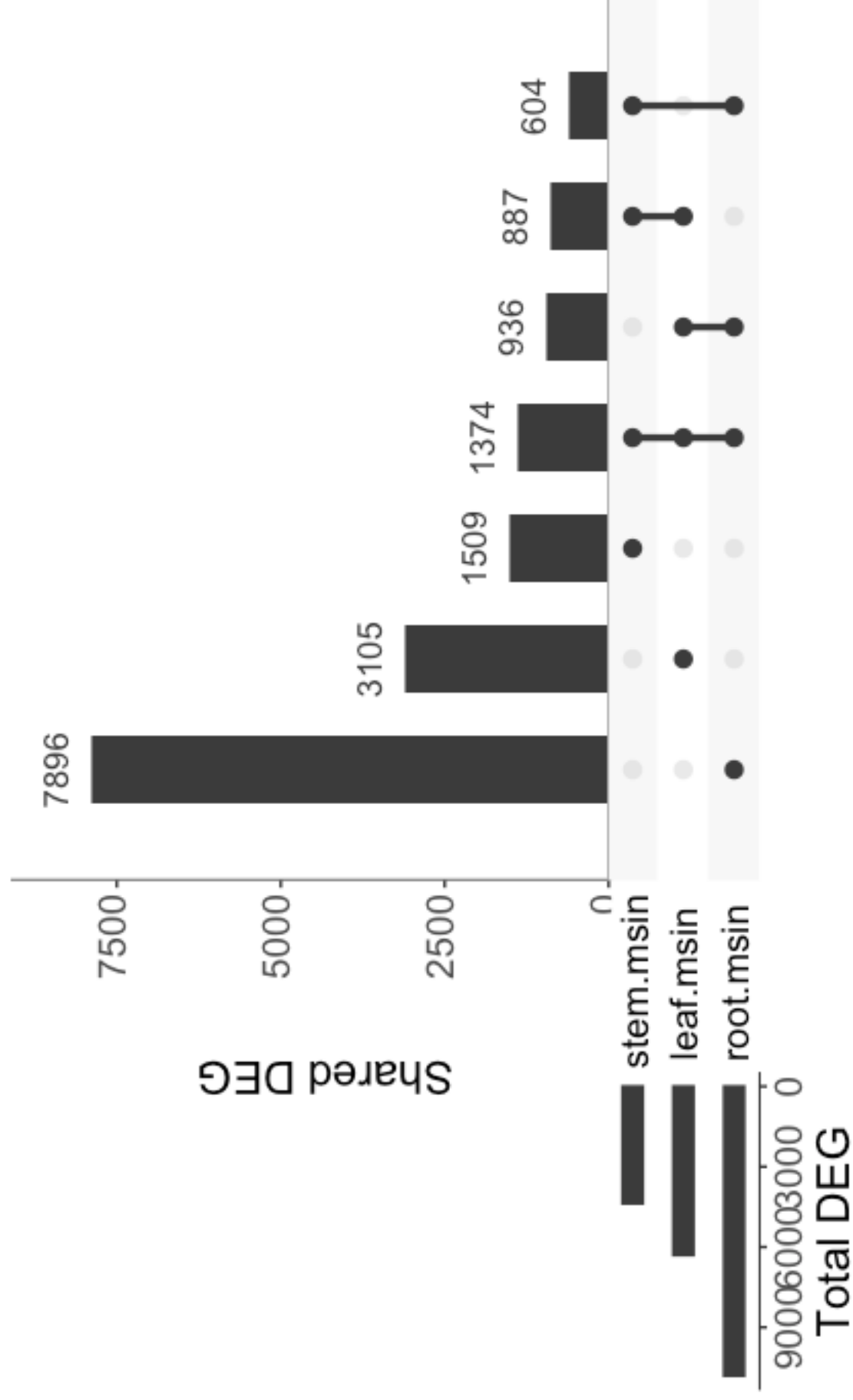

Supplement: Supplementary file 1 — Additional file 1: Figure S1. Number of differentially expressed genes shared between root, leaf and stem tissues between the hybrids and the M. sinensis progenitor. [file 13068_2021_1948_MOESM1_ESM.pdf]

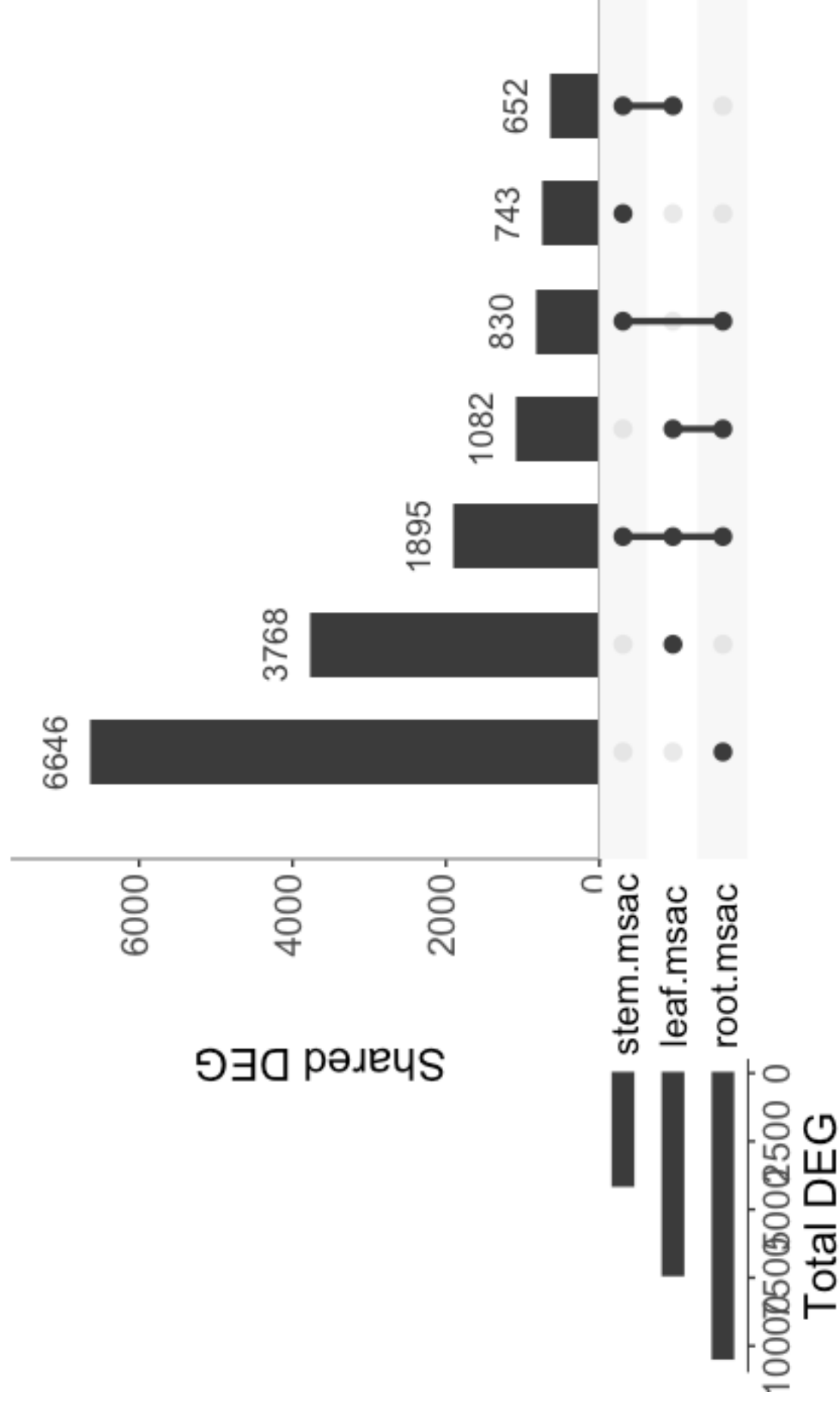

Supplement: Supplementary file 2 — Additional file 2: Figure S2. Number of differentially expressed genes shared between root, leaf and stem tissues between the hybrids and the M. sacchariflorus progenitor. [file 13068_2021_1948_MOESM2_ESM.pdf]

# GO slim term

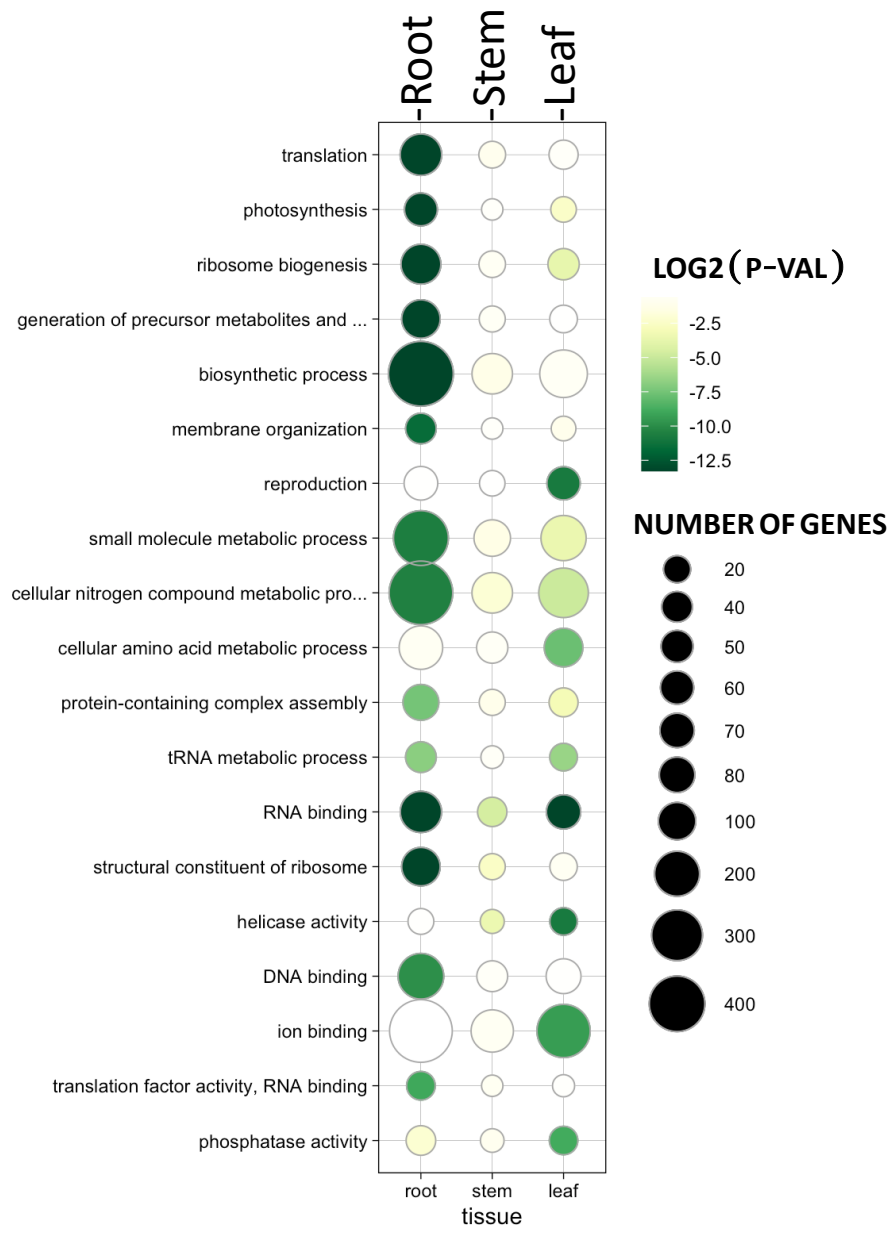

Supplement: Supplementary file 3 — Additional file 3: Figure S3. GO SLIM terms (rows) that were significantly enriched (p < 0.05) in each tissue (columns) among differentially expressed genes (DEG) from the expression analysis between the hybrids and both progenitors. The size of a bubble is proportional to the number of DEG annotated with that GO term. Rows are sorted by descending p-value (F-Fisher test) and the bubble colour is representative to the obtained p-value, from lower (dark green) to higher (light green). Yellow (p > 0.05) and white (p > 0.1) bubbles were not enriched. All the enriched GO SLIM terms for the “biological process” (top 8 rows) and “molecular function” (bottom 5 rows) GO categories were included. [file 13068_2021_1948_MOESM3_ESM.pdf]

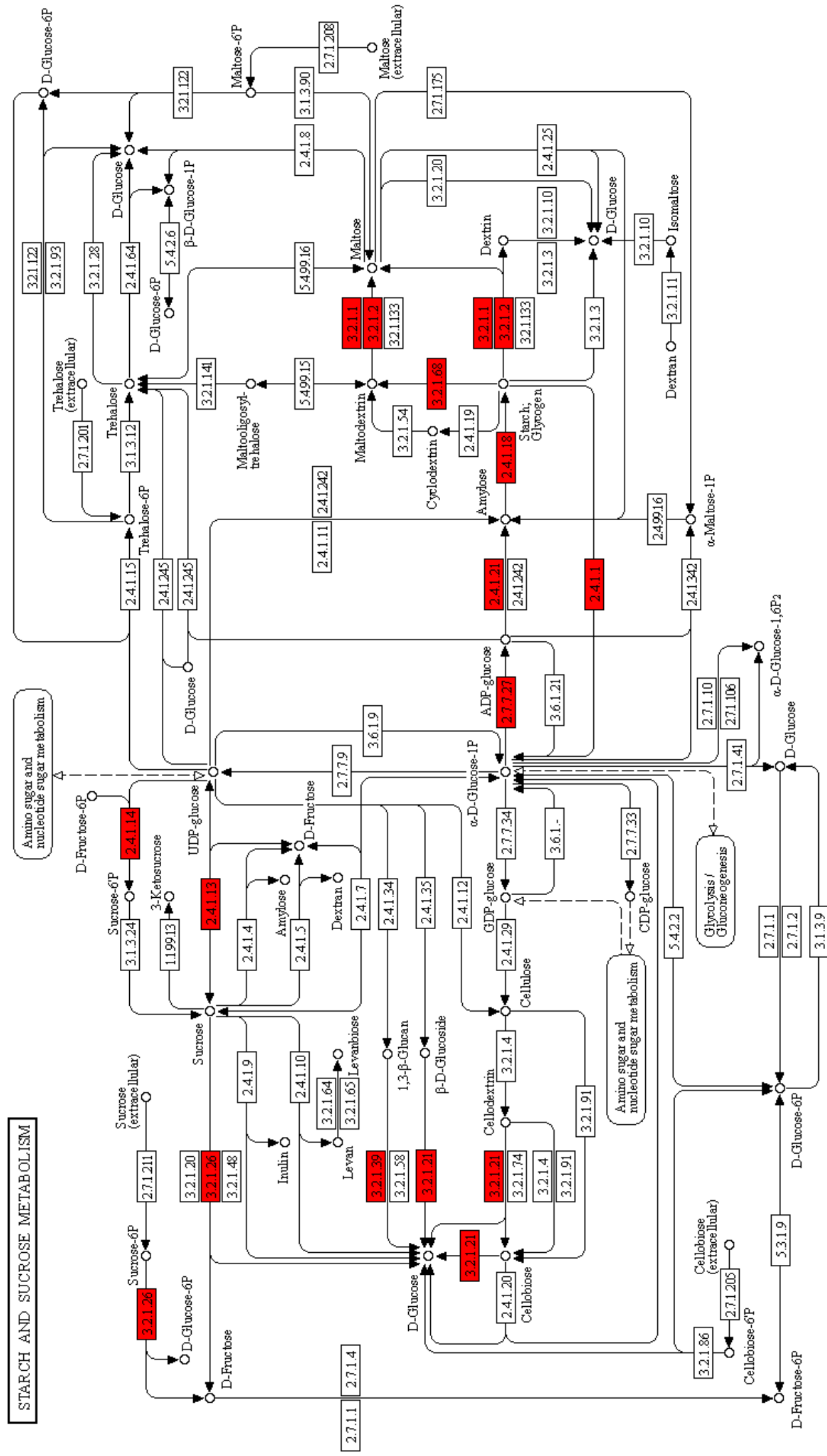

Supplement: Supplementary file 4 — Additional file 4: Figure S4. Down-regulated enzymatic reactions in the “starch and sucrose metabolism” pathway from KEGG (KEGG pathway ath00500) that were down-regulated in “high NSC” hybrids, which had higher concentrations of starch and sucrose. [file 13068_2021_1948_MOESM4_ESM.pdf]

## GLYCOLYSIS / GLUCONEOGENESIS

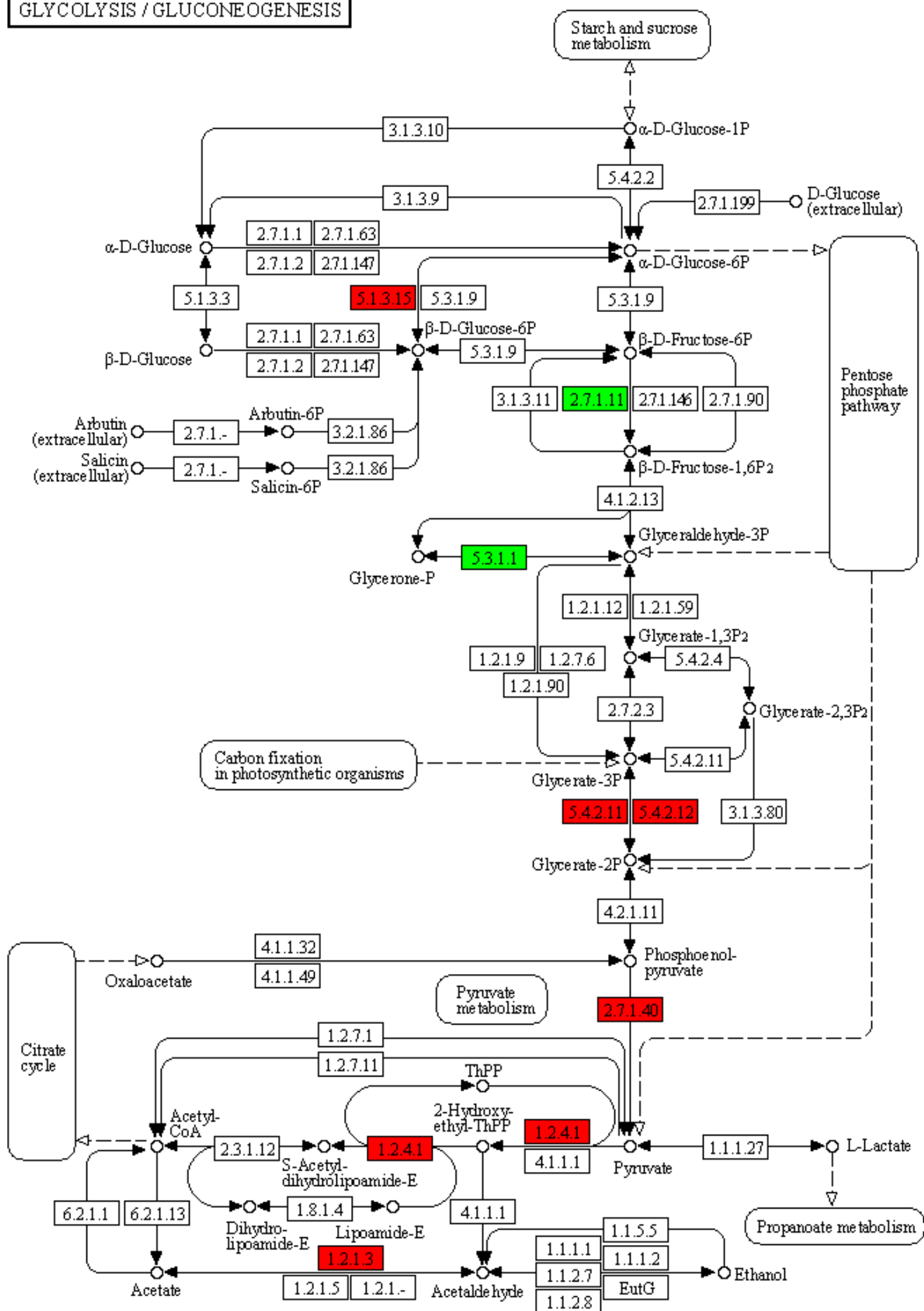

Supplement: Supplementary file 5 — Additional file 5: Figure S5. Enzymatic reactions in the “glycolysis/gluconeogenesis” pathway from KEGG (KEGG pathway ath00010) that were down-regulated (red boxes) or up-regulated (green boxes) in “high NSC” hybrids, which had higher concentrations of starch and sucrose. [file 13068_2021_1948_MOESM5_ESM.pdf]
